# Disseminated Tuberculosis Mimicking as Crohn's Disease in a Paediatric Patient

**DOI:** 10.1155/2023/7312630

**Published:** 2023-06-29

**Authors:** Elizabeth Feenstra, Yentl Driesen, Nicolette Moes, Nathalie Jouret, Koen Vanden Driessche

**Affiliations:** ^1^Paediatric Department, Antwerp University Hospital (UZA), Edegem, Belgium; ^2^Paediatric Department, Ziekenhuisnetwerk Antwerpen (ZNA) Stuivenberg/Jan Palfijn, Antwerp, Belgium; ^3^General Internal Medicine, Infectious Diseases and Tropical Medicine, Antwerp University Hospital (UZA), Edegem, Belgium

## Abstract

Tuberculosis is an important infectious disease for children worldwide. The clinical presentation of tuberculosis in children is diverse and, depending on the affected organs, it is often accompanied with nonspecific symptoms that can mimic other diseases. In this report, we present a case of disseminated tuberculosis in an 11-year-old boy with intestinal followed by pulmonary involvement. The diagnosis was delayed for several weeks due to the clinical picture which was mimicking Crohn's disease, the known difficulties in diagnostic tests and the improvement on meropenem. This case demonstrates the importance of a detailed microscopic examination of gastrointestinal biopsies and the tuberculostatic effect of meropenem which physicians should be aware of.

## 1. Introduction

Tuberculosis (TB) remains one of the most important infectious diseases with an estimated incidence of 10.6 million cases of TB disease and a mortality of 1.6 million people worldwide in 2021 [1]. Children represent approximately 11% of the total burden of TB and 15% of the total mortality. These numbers could, however, be an underestimation of the true burden of paediatric TB due to the difficulty of diagnosing TB, especially in children, and the high mortality without treatment [2, 3]. Infants, young children, and patients with immune deficiencies are susceptible to develop more severe forms of TB such as tuberculous meningitis and disseminated TB. The clinical presentation of TB in children is diverse, and depending on the affected organs, it is often accompanied with nonspecific symptoms that can mimic other diseases.

## 2. Case Report

A 11-year-old boy of Congolese descent, born in Belgium and without significant medical history, presented with nonbloody diarrhoea for several weeks and unintentional weight loss. He reported a recent history of rhinitis and coughing with spontaneous improvement. He received all of his recommended vaccines (BCG vaccine not included) and he never left Belgium. The family history indicated no documented cases of TB or recent visits to a country with a high burden of TB. Physical examination was normal. Laboratory investigations showed a mild microcytic anaemia and slightly elevated inflammatory markers; sedimentation 29 mm/hr (normal range 0–13 mm/hr), and C-reactive protein level 16 mg/L (normal range <3 mg/L). Chest X-ray demonstrated minimally reduced lung translucence with possible ground-glass opacity, but an additional CT-thorax was unremarkable. The tuberculin skin test (TST) and the interferon-gamma release assay (IGRA), executed with T.SPOT.*TB*, were also negative. Abdominal ultrasound demonstrated discrete wall thickening of the terminal ileum with accompanying mesenteric lymphadenitis (four enlarged lymph nodes noted, the largest 27 × 16 mm). One week after presentation, he was referred for a colonoscopy because of markedly elevated faecal calprotectin of 4735 mg/kg (normal range <50 mg/kg) which is indicative of bowel inflammation.

**Figure 1:**
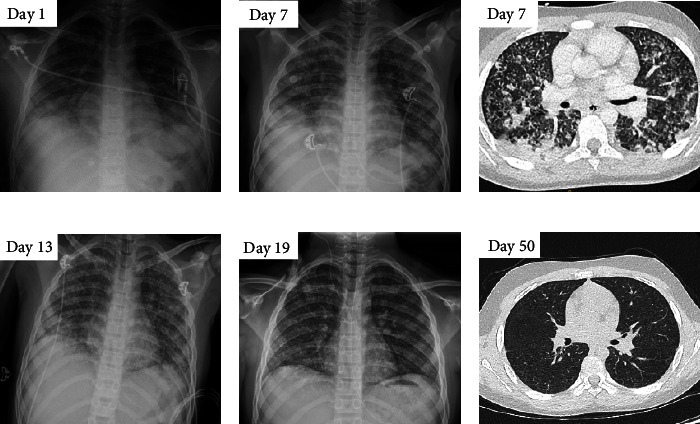
Pulmonary evolution on chest X-ray (CXR) and chest computer tomography (chest CT) during hospitalization and after discharge. (a) CXR on day 1. (b) CXR on day 7. (c) Chest CT on day 7. (d) CXR on day 13. (e) CXR on day 19. (f) Chest CT on day 50.

Eight days after referral, a colonoscopy was performed and ileocecal inflammation was confirmed, a hallmark sign of Crohn's disease. After the procedure, he developed high fever with respiratory symptoms (dyspnea and crackles) and a reduced oxygen saturation of 80%. His chest X-ray (Figure 1(a)) demonstrated bilateral alveolar consolidations and a reticular enhanced lung parenchyma in the lower lobes. Amoxicillin/clavulanate was initiated in assumption of the diagnosis of aspiration pneumonia and respiratory support with low-flow oxygen therapy was started. In the absence of clinical and biochemical improvement, treatment was switched to ceftriaxone and metronidazole on day 5. Further respiratory deterioration was noted on day 7, and respiratory support was switched to high-flow oxygen therapy. A chest X-ray and computed tomography (CT) scan (Figures 1(b) and 1(c)) of the thorax revealed increased asymmetric bilateral (right more than left) patchy alveolar consolidations with adjective ground-glass opacity, suggestive for a superinfection above the initial aspiration pneumonia. One day later, the treatment was switched to meropenem. Eventually, gradual respiratory and biochemical improvement was noted. Respiratory support with high-flow oxygen therapy was tapered and switched to low-flow oxygen therapy on day 14, which could be stopped on day 17. Biopsies of the gastric corpus, the terminal ileum and the ileocecal valve revealed chronic inflammation with granulomas. The pathologist noted that these appeared larger and more abundant than normally encounters in Crohn's disease and TB was in the differential diagnosis. Microscopic examination of the biopsy material was not supplemented by mycobacterial culture (not possible because fixed in formaldehyde) nor PCR analysis. Furthermore, work-up for TB included radiological imaging with a brain MRI and abdominal CT scan. TST and IGRA (QuantiFERON) were repeated. Blood, CSF, gastric lavage (three times), and bronchoalveolar lavage fluid were inoculated for mycobacterial cultures (after fluorescence microscopy). Mycobacterial PCR was performed on CSF and faeces (GeneXpert MTB/RIF® in accordance with the KNCV protocol). Because of persistent gastrointestinal symptoms, despite dietary treatment with Modulen®, corticosteroids were associated together with precautionary TB treatment on day 15. Beside abnormalities revealed on the abdominal CT scan (discrete ileocecal bowel wall thickening, two lymph nodes with calcification and ascites fluid), investigations for TB came back negative. Bacterial cultures and multiplex polymerase chain reaction-test for viral infections and *Pneumocystis jirovecii* on bronchoalveolar lavage fluid were also negative. Faeces were negative for parasites. Meropenem was continued till day 19, and the patient was discharged on day 22. A positive evolution was noted on a new chest CT scan on day 50 (Figure 1(f)), with minor residual abnormalities suggestive of a previous infection. Two months after presentation, the family was screened which demonstrated a positive TST (induration of 15 mm) in his 9-month-old sibling, indicating recent exposure of the family to an infectious TB case. Corticosteroids were tapered to stop, and TB treatment was continued for 9 months. Complete clinical recovery was observed and currently, 12 months after finishing the TB treatment, no symptoms recurred.

## 3. Discussion

Diagnosing TB is often challenging, especially in children, because of the nonspecific symptoms, the difficulties with specimen collection for (microbiological) testing and the low sensitivity of available tests [4]. In this report, we present a probable case of disseminated TB with intestinal involvement and likely also pulmonary involvement. This diagnosis was delayed for several weeks due to the clinical picture which was mimicking Crohn's disease, the known difficulties in diagnostic tests and the improvement on meropenem.

Lymphohematogenous dissemination leading to disseminated TB occurs in 0.5–2% of infected children [5]. Abdominal involvement can be classified according the affected site as lymph node, peritoneal, intestinal, and visceral TB. Intestinal TB is rare and mostly affects the distal ileum and ileocecal valve, followed by the colon and jejunum. The clinical manifestation may lack specificity and include symptoms such as abdominal pain, diarrhoea, and weight loss. Additional suggestive features, such as fever, night sweats, ascites, and lung involvement, may or may not be present. Some histopathological features can favour the diagnosis of intestinal TB, and these have been identified as the presence of confluent and/or larger (>0.05 mm^2^) granulomas, ≥10 granulomas per biopsy site, granulomas with caseous necrosis, and presence of ulcers lined by bands of epithelioid histiocytes and disproportionate submucosal inflammation [6]. Among these features, the presence of granulomas with caseous necrosis is the only specific characteristic of intestinal TB [7]. Additionally, other exclusive indicators of intestinal TB include a positive mycobacterial culture or PCR test and the identification of necrotic lymph nodes on a CT scan. These features exhibit high specificity but demonstrate low sensitivity.

In this case, the respiratory symptoms exacerbated after the general anaesthesia for a colonoscopy. Due to the clinical course, the radiological findings, and the absence of another causative organism, we consider this as possible pulmonary involvement of disseminated TB, but mycobacterial cultures of bronchoalveolar lavage fluid remained negative. Despite administration of general antibiotics, clinical and biochemical improvement was only noted after initiating meropenem. For physicians, this finding can be a misleading argument against the diagnosis of TB. We collected several other publications that report a diagnostic/therapeutic delay due to initial improvement on general antibiotics. Most case reports involved fluoroquinolones [8–12]. In one of these case reports, ciprofloxacin was used in a combination with clarithromycin and linezolid [11]. The same effect has been described with aminoglycosides [13]. We could not find a publication that reports a delay in diagnosis due to the tuberculostatic effect of meropenem. Several studies, however, have been performed and demonstrated the activity of meropenem against *Mycobacterium tuberculosis* [14, 15]. The World Health Organisation approved meropenem together with clavulanate in regimens for patients with multidrug-resistant TB [16].

We consider the positive TST in the 9-month-old brother as circumstantial evidence for TB in our patient. In children, and especially in infants, TB disease and infection indicate a recent or ongoing exposure of TB within a family or community, a so-called sentinel-event.

## 4. Learning Points

Intestinal TB can mimic Crohn's disease. A detailed microscopic examination of gastrointestinal biopsies can help differentiating between intestinal TB and Crohn's disease, for example, large/more abundant granulomas fit better with the diagnosis of TB, as do caseous necrosis. While improvement on antibiotics can be used as an argument against TB, physicians should be aware that this is not uniformly the case because some antibiotics have a tuberculostatic effect. Our report suggests this is also the case for meropenem. Finally, both TB disease and infection in a young family member are sentinel events for recent TB exposure, which can help with establishing the diagnosis.

## Data Availability

The data used to support the findings of this study are included within the article.
